# Outcome of Sleep Rehabilitation in Autistic Children with Sleep Disorders Is Linked to Melatonin Receptor Genes SNPs

**DOI:** 10.3390/ijms26115198

**Published:** 2025-05-28

**Authors:** Elisabetta Bolognesi, Alessandra Carta, Franca Rosa Guerini, Stefano Sotgiu, Cristina Agliardi, Chiara Dettori, Milena Zanzottera, Mario Clerici

**Affiliations:** 1 IRCCS Fondazione Don Carlo Gnocchi ONLUS, Laboratory of Molecular Medicine and Biotechnology, 20148 Milan, Italy; ebolognesi@dongnocchi.it (E.B.); cagliardi@dongnocchi.it (C.A.); mzanzottera@dongnocchi.it (M.Z.); mario.clerici@unimi.it (M.C.); 2 Unit of Child Neuropsychiatry, Department of Medicine, Surgery and Pharmacy, University of Sassari, 07100 Sassari, Italy; carta.ale84@gmail.com (A.C.); stefano.sotgiu@aouss.it (S.S.); chiaradettori06@gmail.com (C.D.); 3 Department of Pathophysiology and Transplantation, University of Milan, 20122 Milan, Italy

**Keywords:** autism, sleep disorders, SDSC scale, melatonin receptors *MT1*, melatonin receptor *MT2*

## Abstract

A significant proportion of children with Autism spectrum disorder (ASD) experience sleep issues, such as insomnia and other disorders, as assessed by the Sleep Disturbance Scale for Children. Our study investigated the link between six single nucleotide polymorphisms (SNPs) in the melatonin receptor genes *MT1* and *MT2* and ASD susceptibility, clinical severity and associated sleep problems. A total of 139 ASD children, 82 siblings, and 53 unrelated healthy controls, all of Sardinian ancestry, were studied; among them, 38 children with co-occurring sleep issues were assessed for the outcomes of a rehabilitative program, including behavioral therapy and sleep hygiene. The *MT2* rs10830963 G allele is more prevalent in ASD children and their siblings compared to the healthy controls, while rs2119882 (*MT1*) and rs1562444 (*MT2*) are associated with DIMS, DA, and SHY. ASD Children carrying the rs2119882 T allele have higher scores for DIMS and DA compared to C allele carriers, and those carrying rs1562444 A allele have higher scores for SHY than G allele carriers. After rehabilitative treatment, homozygous TT carriers of rs2119882 showed less improvement in DIMS symptoms compared to CT and CC carriers. A similar result was observed for AA carriers of SNP rs1562444 about SHY. We may suggest that the *MT1* and *MT2* variants may serve as useful predictive genetic markers for the severity of sleep disorders in children with ASD, potentially informing the design of more targeted rehabilitative treatments.

## 1. Introduction

Autism spectrum disorder (ASD) is a neurodevelopmental disorder that affects communication, social interaction, and behavior [1]. Between 40 and 83% of children/adolescents with ASD suffer from sleep disturbances that include insomnia, resisting bedtime, delayed time falling asleep and maintaining sleep, multiple night and early morning awakenings, among others [2]. The poor quality of sleep can exacerbate abnormal behaviors in ASD children, worsening core symptoms, such as poor social capabilities, restricted repetitive behaviors, and language issues [3]. Moreover, sleep problems in these children can cause the appearance of new behavioral problems, such as hyperactivity, irritability, aggression, anxiety, affective difficulties, attention deficits, and poorer functional and adaptive skills [4,5,6]. Sleep problems in ASD children, thus, can lead to a vicious circle which has a strong impact on the quality of life of the patients and the parents/caregivers [7]. Studies have provided evidence of a relationship between melatonin deficit and social communication impairments in neurodevelopmental disorders. In particular, in ASD patients, a lack of melatonin production was associated with language impairments [8,9]. Other results have showed that nighttime reduced levels of serum melatonin are significantly associated with severe social communication impairments, particularly verbal communication, and imitative play deficit, in children and adolescents with ASD [10,11]. Melatonin supplementation in ASD children, finally, was observed to increase total sleep time and sleep quality, improving behavior and communication [12]. Melatonin is a hormone that regulates sleep, influencing the sleep–wake cycle by the circadian clock and acting as a sleep modulator [13]. Similarly to vitamin D, another hormone suggested to play a role in ASD children [14,15], melatonin is known to be involved in neuroprotection, neuroplasticity, and neurodevelopment through neurotrophic factors [16], and to be endowed with anti-inflammatory and anti-oxidant activities, playing a part also in cardiovascular protection and reproduction and mood disorders [17]. In mammals, the circadian clock is located in the suprachiasmatic nucleus (SCN) of the hypothalamus. The retina, via the retinohypothalamic tract, provides direct information to the SCN on the day/night cycle of the external environment, thereby controlling melatonin synthesis by the pineal gland in response to darkness. Melatonin, in turn, controls the activity of the SCN through a feedback mechanism involving the melatonin receptors 1 and 2 (*MT1* and *MT2*) located in the SCN [18]. Recent studies have showed that the *MT1* and *MT2* receptors are differently expressed in regions involved in rapid eye movement (REM) and non-REM (NREM) sleep. The *MT1* receptors are found in regions involved in the REM phase, whereas the *MT2* receptors are expressed in the NREM region of the reticular thalamus [19], thus differently modulating the two sleep stages. In addition, the *MT1* receptors are highly expressed in the SNC, while the *MT2* receptors are less abundant [20]. Notably, variants in the melatonin receptor genes were associated with insomnia in schizophrenia [21], bipolar disorder [22], non-24-h sleep–wake syndrome [23], and REM sleep latency [24].

We investigated possible associations between six single nucleotide polymorphisms (SNPs) of the melatonin receptor genes, namely rs6847693, rs2119882, and rs6553010 in *MT1* and rs4753426, rs10830963, and rs1562444 in *MT2*, and ASD susceptibility and clinical severity in a cohort of 139 ASD children, 82 ASD siblings, and 53 healthy controls, all of Sardinian ancestry. Furthermore, the possible influence of these polymorphisms on sleep disturbances and the outcome of sleep rehabilitation training, consisting of behavioral therapy and sleep hygiene, was tested in 38 children with ASD who had confirmed sleep problems as measured by the Sleep Disturbance Scale for Children (SDSC) [25].

## 2. Results

### 2.1. Distribution of MT1 and MT2 SNPs Alleles and Genotypes in Children with ASD, Their Siblings and HCs

The distribution of *MT1* rs6847693, rs2119882, rs6553010, and *MT2* rs4753426, rs10830963, and rs1562444 SNPs in children with ASD, their siblings (SIBS), and HCs is reported in Table 1. The genotype frequencies for each polymorphism were in the Hardy–Weinberg equilibrium for the three groups. The results showed that the minor *MT2* rs10830963 G allele is significantly more frequent in ASD children than in HCs (25.0% vs. 14.1% *p* = 0.02; OR: 2.0; 95% CI: 1.1–3.8); this trend is also present for SIBS vs. HC (26.0% vs. 14.1%, *p* = 0.02, OR:2.1; 95% CI: 1.1–4.1). When comparing the genotypes, we found that the frequencies of the rs10830963 genotypes were more skewed in children with ASD than in HCs (*p* = 0.06). However, the dominant model (CG + GG vs. CC) resulted significantly higher in children with ASD than in HCs (CG + GG: 45.0% vs. CC: 26.4%; *p* = 0.02; pc = 0.04; OR: 2.2; 95% CI = 1.0–4.6).

### 2.2. SDSC Scores at T0 in Children with ASD and Their Siblings

Table 2 summarizes the demographic characteristics of the ASD and SIBS enrolled in the study, along with the mean ADOS, CARS, and SDSC scores before the beginning of the trial (T0). All ASD children had scores ranging from subclinical (51–70) to clinically significant (>71) on the total SDSC and its subscales, while their siblings showed scores within the normal range (<50).

### 2.3. Outcome of Rehabilitation on Sleep Disturbances (Behavioral Therapy and Sleep Hygiene)

**Figure 1:**
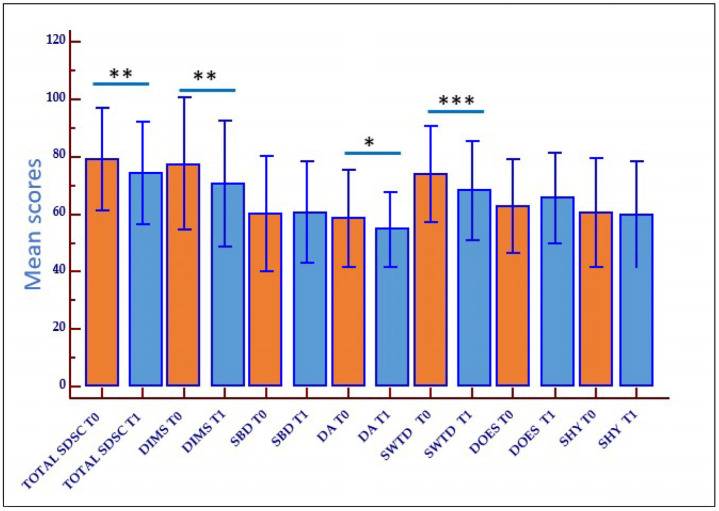
Graph for repeated measures ANOVA for the SDSC scores in children with ASD after rehabilitation treatment. * *p* = 0.04, ** *p* < 0.01, *** *p* > 0.001. T0: pre-treatment, T1: after treatment.

The thirty-eight children with ASD with sleep disorders underwent a 6-month rehabilitation treatment (behavioral therapy for sleep/insomnia disorders): the rehabilitation outcome was measured using the SDSC scale at the beginning (T0) and end (T1) of the trial. After the treatment, the children with ASD had improved their sleep, showing a significant decrease in the mean total scores for the total SDSC (*p* < 0.01), the DIMS (*p* < 0.01), the DA (*p* = 0.04), and the SWTD (*p* < 0.001) at T1. For the SHY item, the decrease in scores was slight and not statistically significant. On the contrary, no improvement was seen for the SBD and DOES subscales, which did not appear to benefit from the rehabilitation treatment (Figure 1).

### 2.4. Effect of MT1 and MT2 Receptors Polymorphisms on Rehabilitation Outcome

#### 2.4.1. Association Between DIMS Scale and the rs2119882 (T/C) Polymorphism of the *MT1* Receptor Gene

At baseline, subjects carrying the rs2119822 T allele polymorphism, which is the least frequent in ASD subjects, were characterized by higher mean DIMS score values than those carrying the C allele (80.7 ± 20.6 vs. 74.7 ± 24.4, respectively). Likewise, subjects with the TT genotype have higher scores (81.6 ± 21.6) than those with the CT (79.8 ± 20.8) or CC (71.2 ± 27.2) genotypes (TT > CT > CC). Despite these observations, the differences were not statistically significant for either alleles or genotypes. Nevertheless, the T allele appears to be linked to more severe symptoms of insomnia in subjects with ASD.

**Figure 2:**
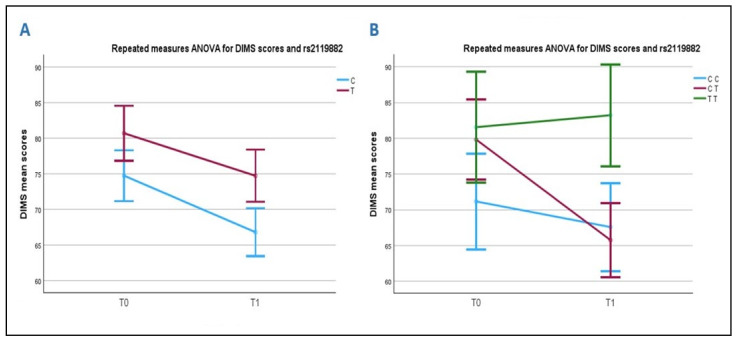
Correlation between rs2119882 and DIMS scores before and after rehabilitation. Chart (**A**): T allele carriers have higher mean scores than C allele carriers. Both improve their sleep symptoms after rehabilitation. Chart (**B**): ASD carriers of the TT genotype have higher pre-rehabilitation scores than CT and CC carriers. After rehabilitation, TT carriers do not improve, whereas CT and CC carriers improve their insomnia symptoms.

No differences with ANOVA repeated measures in the improvement of DIMS symptoms were observed in relation to allelic distribution after treatment (Figure 2A). However, when analyzing the correlation between DIMS scores and genotypes, a significant difference was observed in the rehabilitation outcome, which was dependent on the rs2119882 polymorphism. The differences were significant both between the T0-T1 interval and between groups (*p* = 0.035 and *p* = 0.028, respectively). Notably, subjects carrying the TT genotype worsened after rehabilitation (Figure 2B; green line), whereas those carrying the CT (red line) or the CC (blue line) genotypes showed a positive rehabilitation outcome. Univariate analyses confirmed the significant effect of rs2119882 (*p* = 0.018) on the rehabilitation outcome.

#### 2.4.2. Association Between DA Subscales and the *MT1* Receptor Gene rs2119882 (T/C) Polymorphism

Analogously to what was seen for the DIMS subscale scores, higher baseline scores were present in subjects carrying the rs2119882 T allele compared to those carrying the C allele (63.5 ± 18.3 vs. 54.3 ± 13.9; *p* = 0.015). The mean score of ASD subjects carrying the C allele is similar to that of non-ASD siblings (54.3 ± 13.9 vs. 47.0 ± 3.9).

**Figure 3:**
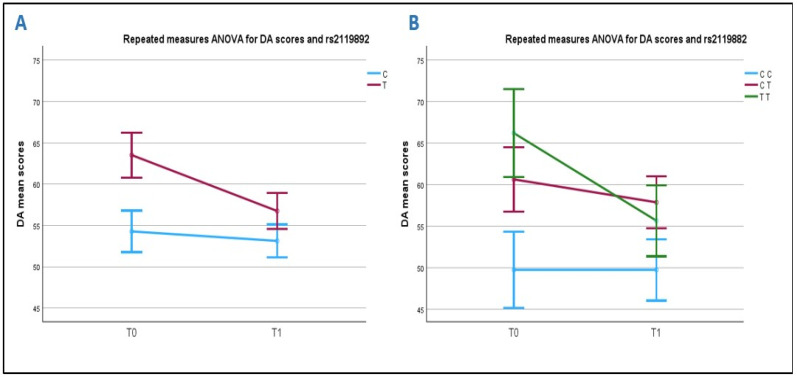
Correlation between rs2119882 and DA scores before and after sleep rehabilitation. Chart (**A**): ASD T allele carriers have higher mean scores than C allele carriers. Both improve their insomnia symptoms after rehabilitation. Chart (**B**): TT genotype carriers have higher scores than CT and CC carriers before rehabilitation. After rehabilitation, TT and CT carriers improve their ASD symptoms, while CC carriers have no mean pathological scores at T0 and T1.

When considering how scores changed after rehabilitation (T0 to T1) in relation to the alleles of the polymorphism, we found that carriers of the T allele significantly improved more than carriers of the C allele, both within subjects (*p* = 0.025, between T allele) and between subjects (*p* = 0.043; T vs. C), even if scores of the T allele carriers at T1 remained higher than those of the C allele carriers (Figure 3A).

Analyzing the association between the DA scale and the rs2119882 genotypes, we observed that carriers of the TT genotype have higher DA values at T0 (66.2 ± 17.7) than those with either the CT (60.6 ± 19.5) or the CC genotype (49.7 ± 4.9). A comparison between CC and TT at time T0 was statistically significant (*p* = 0.006): specifically, carriers of the TT genotype present more severe symptoms in the DA scale. After rehabilitation, the TT and CT carriers improved their sleep symptoms, while the CC carriers had no pathological scores at both T0 and T1. A univariate analysis did not show any association of rs2119882 with the rehabilitation outcome when we used the T0 scores of DA as a covariate (*p* = 0.1) (Figure 3B).

#### 2.4.3. Association Between SHY Score and the *MT2* Receptor Gene rs1562444 (A/G) Polymorphism

**Figure 4:**
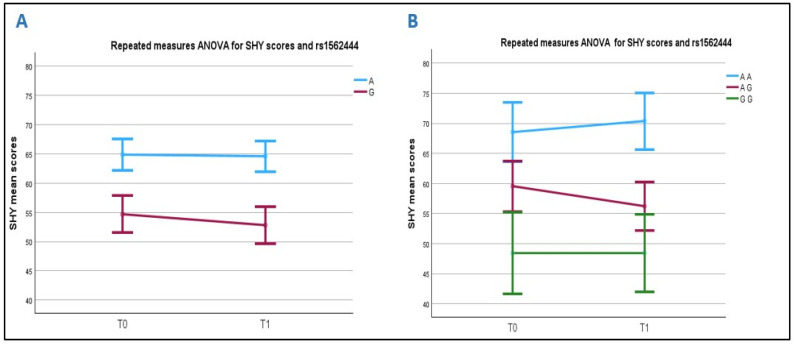
Correlation between rs1562444 and SHY scores before and after rehabilitation. Chart (**A**): T allele A carriers have higher mean scores than G allele carriers. Chart (**B**): The AA genotype carriers have higher pre-rehabilitation scores than AG and GG. After treatment, AA carriers did not improve, AG carriers improved their SHY symptoms only slightly, and GG carriers had no pathological mean scores both at T0 and T1.

The A allele is more frequent in the ASD subjects (57.9%) than in both the siblings (40.6%) and the controls (50.9%), though not significantly (*p* = 0.1 and *p* = 0.3, respectively). Analyzing the SHY subscale scores at time T0 (Figure 4A), we found that the subjects carrying the A allele have significantly higher mean scores than those carrying the G allele (64.9 ± 20.3 vs. 54.7 ± 14.2, respectively; *p* = 0.018) (Table S1). This was confirmed at T1, with the ASD A-allele carriers having a mean score of 64.6 ± 20.2 and the G-allele carriers of 52.8 ± 13.1 (*p* = 0.005).

The genotypes were also associated with different SHY mean scores at both T0 and T1 (*p* = 0.065 and *p* = 0.018, respectively). Specifically, at time T0, significantly higher scores (68.5 ± 22.9) were seen in the ASD subjects carrying the AA genotype compared to those carrying the AG (59.6 ± 15.8) and GG genotypes (48.4 ± 9.1) (AA vs. GG *p* = 0.04).

When analyzing how the scores evolved after rehabilitation, we found that the carriers of the G allele improved, while the carriers of the A allele did not (significant analysis between subjects: *p* = 0.008). Regarding the genotypes, the differences between the subjects were still significant (*p* = 0.031): specifically, higher average values at T1 were seen in the AA genotype carriers (70.4 ± 21.8) compared to those carrying the AG (56.2 ± 15.1) and GG (48.4 ± 9.1), (AA vs.GG *p* = 0.022) genotypes. The AA carriers (blue line) did not show any improvement after rehabilitation, whereas SHY scores normalized in the GG carriers (green line) (Figure 4B). In addition, a univariate analysis validated the significant influence of the rs1562444 (*p* = 0.04) on the rehabilitation outcomes, as we corrected the T0–T1 delta scores for the SHY T0 scores.

No significant correlations were observed between the *MT1* variants (rs6847693 and rs6553010) or the *MT2* variants (rs4753426 and rs10830963) and the total SDSC or its subscale scores at T0, T1 (Table S1), or in the score changes between T1 and T0 (ΔT1–T0). 

We further investigated whether the *MT1* rs2119882 and/or *MT2* rs1562444 risk factors related to sleep disorders in the ASD children at T0 might be associated with the SDSC scale in their siblings, but no correlations were found. On the other hand, as expected, normal SDSC scale values were seen in non-ASD siblings (Table 2).

### 2.5. MT2 rs10830963 (G) Allele, ASD Severity, and Total SDSC and Subscales Scores

Finally, the rs10830963 G allele polymorphism was significantly more frequent in ASD children compared to HCs. No correlations were present between the G allele, the SDSC scales, and ASD severity (ADOS-2, CARS, Leiter, and Wheschler scales) in our study cohort.

## 3. Discussion

To our knowledge, this study is the first to explore the correlation between the severity of sleep disturbances in children with ASD, as assessed by the Sleep Disturbance Scale for Children (SDSC), and SNPs in the *MT1* and *MT2* melatonin receptor genes. We also examined the potential influence of these SNPs on the outcome of a 6-month rehabilitation program, which included behavioral therapy for sleep and insomnia disorders. The results showed that the T allele and TT genotype of the *MT1* receptor (rs2119882) were associated with more severe symptoms on the DIMS and DA subscales, and with a poorer rehabilitation outcome on the DIMS scale. These findings suggest that children with the TT genotype require more intensive rehabilitation treatment. Conversely, no significant improvement after rehabilitation was seen in children with the C allele/CC genotype; the observation that their mean DA score was not pathological (mean score < 50) likely explains this outcome. Therefore, the T allele is a risk factor for the severity of arousal-related sleep disturbances (DA subscale), but it does not influence rehabilitation outcomes. Additionally, the A allele and AA genotype of the *MT2* receptor (rs1562444) were identified as risk factors for more severe symptoms and resistance to rehabilitation, as measured by the SHY subscale (sleep hyperhidrosis).

The rs2119882 polymorphism is located in the promoter region of the *MT1* gene, where it serves as a binding site for multiple transcription factors, thereby regulating receptor expression. The CC genotype of rs2119882 has been significantly associated with insomnia symptoms in schizophrenia within the Korean population [21]. On the Ensembl website for the 1000 Genomes Project, the minor allele frequency of this SNP varies across populations: in East Asian, South Asian, and African populations, the minor allele is the C allele, while in European and American populations, it is the T allele. This suggests that the selective advantage of this promoter variant may differ across populations. As *MT1* receptors are primarily expressed in brain regions associated with REM sleep, our data are consistent with the observed association with DIMS, where REM sleep is a state of high brain arousal during sleep [26,27], and with DA (which includes nightmares and REM parasomnias), as both are intrinsically related to REM sleep disturbances. Sleep hyperhidrosis (night sweating) is another common disorder in children with ASD [28,29]. We found an association between SHY and the rs1562444 SNP, located in the 3′ UTR of the *MT2* gene. Interestingly, the *MT2* receptors are also expressed in the skin and eccrine sweat glands [30]. Melatonin has been shown to influence skin sympathetic nerve activity (SSNA), potentially attenuating mental stress [31].

We also observed a skewed distribution of the rs10830963 G allele in children with ASD and their siblings compared to healthy controls. However, this allele showed no significant correlation with the SDSC scales or ASD severity as measured by clinical and observational scales. This SNP may be in linkage equilibrium with other SNPs involved in sleep disturbances, or that, given the multifactorial genetic nature of ASD, it may influence other pathways. The rs10830963 polymorphism is located in the 5′ promoter region of the *MTNR1B* gene, which may alter mRNA and protein expression [32]. Previous studies have shown increased *MT2* receptor expression and altered melatonin secretion patterns in individuals carrying the G risk allele of rs10830963 [33,34]. In the literature, rs10830963 has been associated with delirium in post-surgical patients [35,36] and with higher fasting blood glucose levels in type-2 and gestational diabetes mellitus in both Caucasian and Asian populations [37,38,39].

Finally, it is worth noting that we did not find significant correlations between the ADOS-2 scores and the SDSC total or subscores. Similarly, we did not observe an influence of cognitive impairment on the prevalence of sleep problems in ASD children [40]. Disorders in initiating and maintaining sleep (DIMS) are a defining feature of insomnia, and are among the most common sleep disturbances in children with ASD [41]. These are often accompanied by subtle alterations in arousal level fluctuations during NREM sleep [42]. Arousal disorders, including parasomnias, can be further classified into NREM-related parasomnias (such as confusional arousals, sleepwalking, sleep terrors, and sleep-related eating disorders) and REM-related parasomnias (including nightmare disorder and REM sleep behavior disorder) [43].

Sleep disorders are often associated with psychiatric comorbidities, such as anxiety, depression, and attention deficits, although a direct correlation with core autism symptoms remains debated [44,45]. The frequent coexistence of ASD and sleep disturbances may be due to reciprocal influences or shared neurobiological roots, such as altered neurodevelopment, clock gene expression, or melatonin metabolism [11,46]. Currently, the relationship between sleep problems and ASD is considered multifactorial, without one being a definitive predictor of the other’s severity. Instead, the association is likely influenced by related psychiatric comorbidities, as is frequently seen in individuals with ASD [6].

Our findings suggest that *MT1* rs2119882 and *MT2* rs1562444 play a role in sleep disorders in ASD children. The T allele of rs2119882 could be a risk factor for more severe symptoms of DA, and is associated with more severe symptoms of DIMS, though not significantly. Furthermore, the TT genotype appears to negatively impact rehabilitation outcomes, indicating a need for more intensive, individualized treatment for these subjects, particularly for DIMS. Similarly, for sleep hyperhidrosis, the A allele/AA genotype of *MT2* rs1562444 may be a risk factor for more severe symptoms and resistance to rehabilitation, while the G allele/GG genotype may offer some protection. Identifying the predictive biomarkers for sleep disorders in ASD children is crucial for tailoring personalized rehabilitative interventions aimed at improving the quality of life for children with ASD and their families, and for understanding the complex interaction between sleep and neurodevelopment.

### Limitations of the Study

This pilot study was conducted with a relatively small cohort of children with ASD and concomitant sleep disorders, which may limit the strength of the results; a larger panel of ASD children is needed to confirm our results. Several factors contribute to this limitation.

First, our Child Neuropsychiatry Division at the University Hospital of Sassari is one of only two referral centers for ASD in northern Sardinia, and we primarily treat both outpatient and inpatient cases. However, for this study, we decided not to include inpatient cases to avoid introducing bias from more severe cases of autism, particularly those requiring MRI due to additional neurological or syndromic symptoms. This decision may have restricted the generalizability of our findings, as milder cases of ASD may not have been adequately represented.

Second, Sardinia is the least populated region of Italy, with a population of approximately 1.5 million residents and the lowest birth rate in the country. This demographic factor may explain the relatively small number of participants in our study. Nonetheless, the sample remains a good representative of the Sardinian pediatric population affected by autism.

Third, Sardinia is ethnically and genetically homogeneous. The genetic analysis in this study was restricted to individuals of Sardinian ancestry, which may limit the applicability of our findings to other ethnic groups. A replication study among continental Italian ASD children should be conducted to confirm our results. Despite this limitation, we believe our study provides a valuable starting point that warrants further investigation in larger cohorts of children with ASD from mainland Italy and other ethnic backgrounds.

Another limitation is the sample size for secondary scales. In the analysis of secondary SDSC scales, we observed significant symptom improvement for all items, except for the DIMS and SBD subscales, which did not show benefits from the rehabilitation treatment. This could be attributed to the small sample size of patients undergoing the sleep rehabilitation treatment, which limits the statistical power to detect changes in these subscales.

## 4. Materials and Methods

### 4.1. ASD Patients, Siblings and Healthy Controls

A total of 139 children (102 males, mean age 9.2 ± 3.6 years), all of Sardinian ancestry, with a diagnosis of ASD according to the DSM-5 criteria [1] and their 82 non-autistic siblings (47 males, mean age 9.8 ± 4.3 years) were enrolled at the Child Neuropsychiatry Division, University Hospital of Sassari (Italy). All cases were retrieved from our outpatients’ repository. Extensive neuropsychological and behavioral analyses were performed. The global cognitive status was evaluated by the Leiter Intelligence Scale [47], the Wechsler Intelligence Scale [48], and Raven’s Progressive Matrices [49]. Diagnostic tools to measure the clinical severity of symptoms included the Autism Diagnostic Observation Schedule 2 (ADOS-2) [50], the semi-structured parent’s interview Autism Diagnostic Interview-Revised (ADI-R) [51], and the Childhood Autism Rating Scale (CARS) [52]. Inclusion criteria included the following: (a) children aged between 3 and 12 years; (b) a primary diagnosis of ASD; and (c) the results of the ADOS-2 test that scores the symptoms of autism. Exclusion criteria were a diagnosis of psychotic disorders and/or intellectual disability, and/or other developmental disabilities according to the DSM-5 criteria. Patients with an ascertained lesion of the CNS and/or a genetic syndrome were also ruled out from the study.

Among the 139 children with ASD, 38 subjects (27.3%; 25 males, mean age 13.3 ± 1.6 years) referred to suffer from sleep disturbances, underwent 6 months of rehabilitation and behavioral therapy for their sleep disorder. These 38 children were assessed with the Sleep Disturbance Scale for Children (SDSC) by Bruni et al. [25], both at the beginning (T0) and at the end (T1) of the sleep rehabilitation treatment to monitor its efficacy. Among the 82 healthy siblings, 15 (18.3%; 4 males, mean age 13.3 ± 2.3 years) were assessed by the SDSC and were enrolled as controls. The SDSC is a rating scale that analyzes the rate of occurrence of sleep disorders in children aged 3 to 16 years. It is a parent-reported assessment tool based on 26 items with a 5-point Likert scale, ranging from 1 (never) to 5 (always). The SDCS is divided into six subscales: DIMS, disorders initiating and maintaining sleep; SBD, sleep breathing disorders; DA, disorders of arousal; SWTD, sleep–wake transition disorder; DOES, disorders of excessive somnolence; SHY, sleep hyperhidrosis. The total score is calculated by summing the scores of each subscale, ranging from 26 to 130. The score can be classified as: 0 = normal scores (<50), 1 = subclinical scores (51–70), 2 = clinically significant scores (>71). Clinical evaluation was blinded to the *MT1*/*MT2* genotypes of the subjects.

Healthy controls (HCs) included 53 Sardinian adult subjects (16 males and 37 females, 35.1 ± 5.2) with no sleep issues. Although no gender difference is reported for the frequency of the SNPs analyzed, we preliminarily performed a regression analysis and confirmed no relationship with sex. Age matching was not used, as only genetic data were used to compare children with ASD, their siblings, and HCs.

This study was designed and conducted according to the Declaration of Helsinki; the research protocol was approved by the Don Gnocchi Foundation Ethical Committee (protocol n. 06_18/05/2016) on 18 May 2016.

### 4.2. Melatonin Receptors SNPs Genotyping

Venous blood in EDTA or saliva was collected from children with ASD and their siblings. Genomic DNA from blood was obtained using a standard phenol/chloroform procedure; whereas, for saliva, the ORAgene-DNA (DNA Genotek Inc., Ottawa, ON, Canada) was used. Genomic DNA from the HCs was extracted from saliva collected with Norgen’s Saliva DNA Collection and Preservation Devices using Norgen’s Saliva DNA Isolation Reagent Kit (Norgen Biotek, Thorold, ON, Canada).

The *MT1* rs6847693, rs2119882, rs6553010, and *MT2* rs4753426, rs10830963, and rs1562444 SNPs were estimated by Real-time Allelic discrimination using TaqMan Assay probes (Applied Biosystems, Carlsbad, CA, USA), C_26399857_20, C_16100974_10, C_11782809_10, C_289583_10, C_3256858_10, and C_8369474_10, respectively.

Each reaction was performed in a 10 µL volume as follows: 1 µL of DNA/sample at the concentration of 10 ng/µL, 0.25 µL of 40X probe, 5.0 µL of TaqMan Genotyping Master Mix (Applied Biosystems, Carlsbad, CA, USA), and 3.75 µL of DNAse-free water. Experiments were performed on 96-well plates, and amplification was performed on a CFX96^TM^ System (Bio-Rad, Hercules, CA, USA). The PCR consisted of a hot start at 95 °C for 10 min, followed by 40 cycles of 94 °C for 15 s and 60 °C for 1 min. Fluorescence detection took place at 60 °C. In each experiment, control samples of known genotypes and a negative control were included. After amplification, an Allelic discrimination plot was generated by the software, showing homozygote clusters, heterozygote clusters, and the negative controls, allowing for genotyping of the samples.

### 4.3. Statistical Analysis

The Hardy–Weinberg equilibrium (HWE) for the *MT1* rs6847693, rs2119882, and rs6553010, and the *MT2* rs4753426, rs10830963, and rs1562444 SNPs was calculated using a chi-squared method in both cases and controls. The 2 × 2 contingency tables were used to compare the distribution of the SNP alleles between children with ASD, their siblings, and HCs. Comparisons of the genotype distributions between groups were conducted using 2 × 3 contingency tables to assess the differences in the six-genotype distributions across ASD individuals, their siblings, and control subjects (ASD vs. controls, ASD vs. siblings, and siblings vs. controls). When a significant *p*-value was found after the Bonferroni correction for 2 degrees of freedom (DF), a 2 × 2 contingency table was applied and the odds ratio (OR) and its 95% confidence interval (CI) were used to measure the association of each polymorphism/haplotype with the disease. The *p*-value was considered significant when <0.05 after the Bonferroni correction for the proper degrees of freedom (Pc). The associations among ASD and *MT* receptor polymorphisms with scores of clinical, behavioral, and functioning scales were tested with the parametric ANOVA or non-parametric Kruskal–Wallis and Mann–Whitney tests, depending on the fitness of the scales to the normal distribution measured with the non-parametric Kolmogorov–Smirnov test.

An analysis of the changes of all the total SDSC scores and SDSC subscales (DIMS, SBD, DA, SWTD, DOES and SHY) scores after the treatment (T0 vs. T1) and their correlation with the genotypes of the melatonin receptors were assessed by repeated measures ANOVA. To confirm the lack of relation with sex, an univariate analysis of the SNP association was applied considering the SDSC score as dependent variable, SNPs as independent variable, and sex as covariate.

Data were analyzed by SPSS version 29.0 (IBM Corp., Armonk, NY, USA), the open source openEpi (https://www.openepi.com (accessed on 1 February 2025), and the R version 4.3.3 (29 February 2024 ucrt) softwares.

## 5. Conclusions

This pilot study suggests a direct association between the rs2119882 *MT1* and rs1562444 *MT2* SNPs and sleep disorders, specifically DIMS, DA, and SHY in children with ASD. The T allele of rs2119882 may be considered a risk factor for more severe symptoms of DA, while the A allele of rs1562444 appears to be linked with more severe SHY disorders.

Moreover, the rs2119882 TT genotype is associated with poorer rehabilitation outcomes for DIMS, while the AA genotype of rs1562444 is associated with a lower likelihood of recovery from SHY disorders following rehabilitation.

Based on these preliminary findings, the rs2119882 *MT1* and rs1562444 *MT2* variants could serve as valuable predictive genetic markers for the severity of sleep disorders in ASD children. These markers may help guide the development of more personalized, targeted treatments to improve outcomes for affected patients.

## Figures and Tables

**Table 1:** Allele and genotype distributions of *MT1* rs6847693, rs2119882, and rs6553010 and *MT2* rs4753426, rs10830963, and rs1562444 in ASD children, their siblings (SIBS), and healthy controls (HCs).

	Sardinian ASD		ASD vs. SIB	ASD vs. HC	Sardinian SIBS		SIB vs. HC	Sardinian Healthy Controls	
	N	%	*p*	*p*	N	%	*p*	N	%
*MT1* rs6847693									
T	250	90.0			148	90.0		95	89.6
C	28	10.0	0.9	0.9	16	10.0	0.8	11	10.3
*MT1* rs2119882									
C	160	58.0			95	58.0		54	50.9
T	118	42.0	0.9	0.2	69	42.0	0.3	52	49.6
*MT1* rs6553010									
G	241	87.0			140	85.0		91	85.9
A	37	13.0	0.7	0.8	24	15.0	0.9	15	14.1
*MT2* rs4753426									
C	139	50.0			89	54.0		47	44.4
T	139	50.0	0.4	0.3	75	46.0	0.1	59	55.6
*MT2* rs10830963									
C	209	75.0			122	74.0		91	85.9
G	69	25.0	0.8	0.02	42	26.0	0.02	15	14.1
*MT2* rs1562444									
A	144	52.0			80	49.0		54	50.9
G	134	48.0	0.5	0.8	84	51.0	0.7	52	49.6
N	278				164			106	
*MT1* rs6847693									
C/C	1	1.0			1	1.0		0	0.0
T/C	26	19.0			14	17.0		11	20.8
T/T	112	80.0	0.8	0.8	67	82.0	0.8	42	79.2
*MT1* rs2119882									
C/C	51	37.0			28	34.0		15	28.3
C/T	58	41.0			39	48.0		24	45.3
T/T	30	22.0	0.7	0.5	15	18.0	0.5	14	26.4
*MT1* rs6553010									
A/A	2	1.0			1	1.0		0	0.0
G/A	33	24.0			22	29.0		15	28.3
G/G	104	75.0	0.9	0.6	59	72.0	0.7	38	71.7
*MT2* rs4753426									
C/C	37	27.0			23	28.0		9	17.0
C/T	65	46.0			43	52.0		29	54.7
T/T	37	27.0	0.5	0.4	16	20.0	0.2	15	28.3
*MT2* rs10830963									
C/C	77	55.0			46	56.0		39	73.6
C/G	55	40.0			30	37.0		13	24.5
G/G	7	5.0	0.7	0.06	6	7.0	0.08	1	1.9
*MT2* rs1562444									
A/A	39	28.0			21	26.0		13	24.5
A/G	66	47.0			38	46.0		28	52.9
G/G	34	25.0	0.8	0.8	23	28.0	0.7	12	22.6
N	139				82			53	

**Table 2:** Demographic and Clinical Characteristics of the ASD and SIBS Groups.

	ASD	SIBS	
	N = 38	N = 15	*p*-Value
AGE [years] *	13.3 ± 1.6	13.3 ± 2.3	0.5
SEX (% males)	65.7	26.7	0.15
ADOS *	11.47 ± 4.8	N.T.	-
CARS *	39.6 ± 7.9	N.T.	-
TOTAL SDSC *	79.24 ± 17.7	42.5 ± 3.3	<0.001
DIMS *	77.5 ± 22.9	41.5 ± 1.8	<0.001
SBD *	60.24 ± 20.0	45.5 ± 1.9	0.009
DA *	58.53 ± 16.7	47.0 ± 0.0	0.017
SWTD *	74.0 ± 16.6	45.8 ± 5.6	<0.001
DOES *	62.9 ± 16.4	44.5 ± 3.2	<0.001
SHY *	60.6 ± 18.7	45.6 ± 1.7	0.006

Legend: * Mean ± standard deviation; N.T.: Not Tested.

## Data Availability

The data presented in this study are available upon request from the corresponding author.

## Supplementary Materials

The following supporting information can be downloaded at: https://www.mdpi.com/article/10.3390/ijms26115198/s1.
